# Editorial: Sulforaphane and isothiocyanates in health

**DOI:** 10.3389/fnut.2025.1558025

**Published:** 2025-02-11

**Authors:** Jed W. Fahey, Diego A. Moreno, Paul Licciardi, Francesco Grassi, Tom C. Karagiannis, Nadia Mazarakis

**Affiliations:** ^1^Departments of Medicine, Pharmacology and Molecular Sciences, Psychiatry and Behavioral Sciences, and iMIND Institute, School of Medicine, Johns Hopkins University, Baltimore, MD, United States; ^2^Institute of Medicine, University of Maine, Orono, ME, United States; ^3^Phytochemistry and Healthy Food Lab (LabFAS), CSIC, CEBAS, Campus Universitario de Espinardo, Murcia, Spain; ^4^Murdoch Children's Research Institute, Royal Children's Hospital, Melbourne, VIC, Australia; ^5^Department of Paediatrics, The University of Melbourne, Parkville, VIC, Australia; ^6^SSD Lab RAMSES, IRCCS Rizzoli Orthopaedic Institute, Bologna, Italy; ^7^The University of Melbourne, Parkville, VIC, Australia

**Keywords:** glucosinolate, myrosinase, clinical, epidemiologic, chronic disease, antimicrobial, food, phytochemical

The capacity of isothiocyanates (ITC) such as sulforaphane (SF; 4-methylsulfinylbutyl-ITC) to be of clinical utility in preventing and treating chronic disease is clear. The health-promoting, preventive, protective, and therapeutic applications of SF are well established, having been the subject of thousands of studies including more than 125 clinical trials. This, despite the fact that as a natural compound present in food, SF cannot and should not be positioned as a drug, nor is it within the province of the pharmaceutical industry. The many mechanisms of action of SF in mammalian systems have been extensively documented. It is the most potent naturally occurring inducer of the Keap1-Nrf2 pathway, which is best-known for its upregulation of antioxidant and detoxification mechanisms as well as its anti-inflammatory potency.

**Figure 1:**
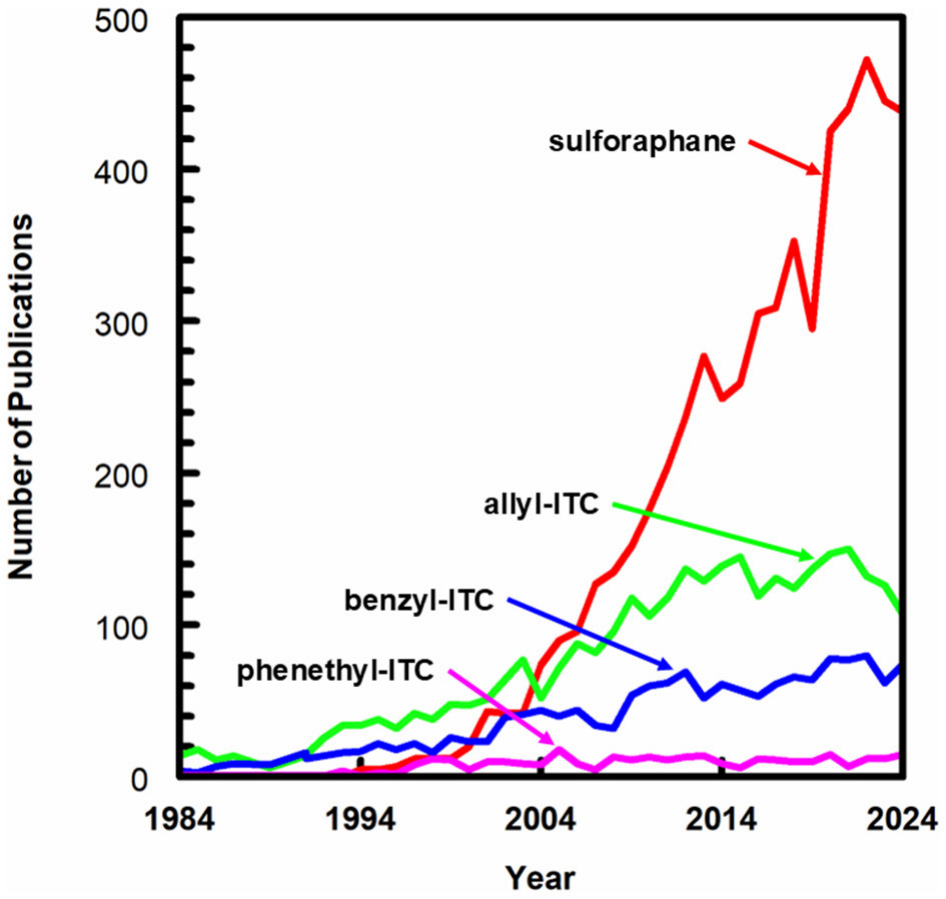
Annual number of peer-reviewed papers on SF, allyl-, benzyl-, and phenethyl- ITC over the last 40 years. Data for graph derived from a Boolean search of Scopus^®^ on Jan 3, 2025.

The establishment of health claims *per se* is problematic from a regulatory perspective since SF and other plant-sourced phytochemicals (a.k.a. phytonutrients or bioactives) are omnipresent in non-ultraprocessed plant foods. SF or its biogenic precursor, glucoraphanin can also be delivered in standardized and/or enriched supplement form. As becomes clear in the contributions for this Research Topic, research interest in ITCs has continued to grow (Figure 1) since the discovery of SF's biological activity the early 1990s (2). More and more applications to a wide variety of diseases continue to be investigated despite lack of regulatory clarity and in the absence of the massive pharmaceutical funding which drives so many other clinical investigations.

Although much of the research on SF and other ITCs is associated with its ability to activate the Keap1-Nrf2 pathway, it exhibits a range of other important biological effects (e.g., inflammation modulation through NF-κB downregulation, infection control, immune system support, selective antibiosis, and cell cycle control), in preclinical and clinical interventions. The dose-response data span disease states and tissue types and indicate that biologically relevant quantities of SF and other isothiocyanates can be provided with practical food- or supplement-based delivery systems. Other aspects of their bioavailability, including potential synergistic, additive, or antagonistic effects coming from combined treatments or food matrix effects are not yet as well understood at the clinical level.

This Research Topic brings together new ideas and provide an updated consensus on the bioactive SF, and other structurally-related and biologically active ITCs. We have thus brought together two reviews (Ramakrishnan et al. and Wang et al.), one epidemiologic study (Liu et al.), two mechanistic studies (Plafker et al., Zimmerman et al.), two clinical studies (Giron et al. and Steenwijk et al.), and a hybrid study in which both animal models and a human longitudinal study are combined (Yang et al.).

Giron et al. conducted the first ever study of the effects of SF on human beings living with HIV. In their 16-week pilot study of 14 virally suppressed HIV patients, given 225 μmol SF daily, a reduction in C-reactive protein was demonstrated. This should encourage more clinical work to examine the efficacy of ITCs not just on people living with HIV, but with other chronic conditions and diseases. The review by Wang et al. is therefore extremely timely in that they evaluated synergisms between dietary ITC and anticancer agents. The word “synergy” has been greatly misused in both the scientific and popular literature. Applying the classic definition of synergy (1), SF is truly synergistic with a lengthy list of drugs and other natural products and the mechanism(s) of those synergies are actually becoming well understood. The review in this Research Topic nicely explains some of these synergies and their mechanisms as they pertain to cancer therapeutic drugs.

The effects of broccoli consumption on a variety of outcomes, primarily cancer-related, have been examined in a number of prospective cohort studies over the years. To our knowledge however, Liu et al. are the first to assess the effects of the frequency of broccoli consumption on all-cause (and cause-specific) mortality. Their findings, based on National Health and Nutrition Examination Survey (NHANES) data from 12,486 adults, confirmed that broccoli consumption 1–2 times per week was associated with a 32–43% lower all-cause mortality risk.

Neurodevelopmental and neurodegenerative conditions that are impacted by treatment with ITCs are extensively reviewed by Ramakrishnan et al. who give special scrutiny to the >80 preclinical studies and 16 clinical studies evaluating the effects of SF on autism spectrum disorder (ASD) and schizophrenia. ASD is also the focus of an exciting new animal model and human clinical study following 12 weeks of SF (equivalent to 30 μmol) supplementation (Yang et al.). Changes in abundance of specific microbial taxa were associated with improvements in ASD symptoms following SF treatment in both rats and humans.

In a 12-subject preliminary intervention, Steenwijk et al. showed that when a single oral intake of SF in the form of broccoli sprouts (16 g) was followed by a high-calorie challenge, platelet responsiveness and improved functionality. These improvements have important clinical promise for safe and efficacious platelet-targeting phytotherapeutics that could have dramatic implications in a variety of thrombotic and chronic inflammatory conditions.

Allyl-ITC, most abundantly found in mustards, white cabbage, radish, and wasabi, has already shown great promise experimentally against bladder cancer. It has long been known to have an array of direct *in-vitro* antimicrobial potencies, thus leading to great interest in its use in food packaging systems. Zimmerman et al. have now provided dramatic data on the antimicrobial properties of allyl-ITC in a *Drosophila* model system. This demonstration of concentration-dependent direct antimicrobial properties as well as its ability to modulate host production of antimicrobial peptides adds to the dossier of allyl-ITC functionality which may have broader applicability to the ITCs in general.

And finally, in a sophisticated model of human retinal pigment epithelial cells, Plafker et al. provide evidence that 25 μM SF elicits cellular responses consistent with its being a fasting/caloric restriction mimetic. Increased mitochondrial mass, reduced glucose uptake, and suppression of insulin signaling are among the modes of action identified, pointing to new plausible mechanisms for SF effect under conditions of metabolic stress (i.e., hyperglycemia, mitochondrial dysfunction) and calling for this theory to be tested promptly in animal models.

Overall, the extensive scope of this Research Topic on SF and other ITCs emphasizes their potential utility in preventing and managing a wide range of human pathologies. While certain mechanisms of action, particularly induction of the Keap1-Nrf2 pathway, are well established, further exploration of molecular mechanisms is required. In this context, there is accumulating evidence indicating epigenetic mechanisms, including DNA methylation and histone modification, associated with SF. Further exploration of genetic and epigenetic modifications may enhance our understanding of the underlying mechanisms accounting for the broad beneficial health effects of SF and other ITCs, as highlighted in this Research Topic.
